# Championing survival: connecting the unknown network of responders to address out-of-hospital cardiac arrest

**DOI:** 10.1186/s13049-020-00748-3

**Published:** 2020-06-03

**Authors:** Ronan McBride, Chantal F. Ski, David R. Thompson, Tom Quinn, Mark H. Wilson

**Affiliations:** 1 Ronan McBride, Anaesthetics, Theatres and Intensive Care Services, Southern Health and Social Care Trust, Portadown, UK; 2 School of Nursing and Midwifery, Queen’s University Belfast, 97 Lisburn Rd, Belfast, UK; 3 Emergency, Cardiovascular and Critical Care Research Group, Kingston University London and St George’s, London, UK; 4 Department of Neurosurgery, Imperial College London, London, UK

**Keywords:** Out-of-hospital cardiac arrest, Emergency medical services

## Abstract

Early intervention for out-of-hospital cardiac arrest (OHCA) presents a challenge for Emergency Medical Services (EMS) across Europe. Strategies designed to address this include education and training initiatives for citizens and building CPR skills capacity and awareness amongst health care professionals. However, there is a need to improve access to volunteer first responders who can commence CPR and defibrillate before the arrival of EMS. In the UK, initiatives such GoodSAM have integrated crowdsourcing technology with ambulance services to allow them autonomy in alerting responders to OHCAs which is parallel to an EMS dispatch. These services are building capacity to improve the initial ‘call for help’ and time to commence CPR and defibrillation if indicated. The next step is to identify and implement appropriate methods for public engagement, involvement and eventual networking of resources with statutory bodies such as local EMS. As crowdsourcing volunteer responders is at an early stage, there is a need to determine whether crowdsourcing is associated with patient outcomes, what its impact is on those responding to OHCA, whether it facilitates or impedes current services, and whether it is a safe and cost effective way to involve citizens to intervene in the community during cardiac arrest or other medical emergencies? Addressing such issues is likely to provide further insight into the role and effectiveness of new technologies and their potential impact on the wider community.

## Background

Intervening early in an out-of-hospital cardiac arrest (OHCA) is an ongoing challenge for Emergency Medical Services (EMS). There are many factors influencing the disparity in outcomes across Europe, and core approaches to improve survival rates are set out in the ‘Chain of Survival’ with early recognition, early CPR, early defibrillation and effective post resuscitation care being paramount [1]. The median incidence of Return of Spontaneous Circulation (ROSC) across 27 European countries is 30% [2], but ranges from as high as 50% to as low as 9%. In the UK, survival to hospital discharge from an OHCA is at best around 12% in England [3] and 10% in Northern Ireland [4].

In Northern Ireland, Emergency Medical Services attend over 1400 OHCAs per year [4]. Approximately 60,000 people suffer an OHCA each year in the UK with around half (28,000) having CPR attempted by EMS. ROSC at hospital handover from EMS is between 13 and 27% and reported survival to hospital discharge is between 2.2 and 12% in the UK [3]. These stark and frankly depressing outcomes and statistics demonstrate an urgent need to identify and evaluate novel approaches to improving survivability of OHCAs.

## What is happening now

Refining ways of dealing with OHCAs and training large cohorts of citizens across the UK are popular approaches to improving the chances of bystander-initiated CPR. Each of the UK nations have published their own resuscitation strategies [5–7] and within each the current drive is to improve all aspects of the Chain of Survival, with a focus on key components such as early recognition and ‘call for help’, early CPR and early defibrillation [1].

Enhancing resuscitation education and its application is a high priority [8]. In England, CPR training has been added to the school curriculum from 2020 in order to embed CPR awareness, knowledge and skills in the citizens of tomorrow. The British Heart Foundation has been delivering ‘Restart a Heart Day’, which is now a global initiative delivered together with multiple statutory organisations and charitable bodies, successfully training large portions of the population in CPR: in the UK over 238,000 were trained in 2018 alone [9]. Increasing the number of those trained in CPR is an approach that is recognised as a pathway to improve ‘quality and timeliness of treatment’ in out of hospital cardiac arrests [10].

A valuable resource that has been of great support in medical emergencies in the community is Community First Responder Schemes (CFRs). Many ambulance services across the UK and Ireland have community groups that are supplementary services delivering lifesaving treatment to those in critical need. For example, in the Republic of Ireland, approximately 600 CFRs are operational across the country [11]. These resources however are finite, covering a large geographical area and have variances in the levels of support and training they receive as they can be either integrated or independent from the EMS [12].

Other organisations focus on specific groups to build CPR skills capacity and awareness. The Resuscitation Council (UK) provides high quality CPR and automated external defibrillator (AED) training to health care professionals across the UK. In 2016–2017, nearly 70,000 people were trained in immediate life support (ILS) and over 22,000 were trained in advanced life support (ALS) [13]. This is a huge cohort of trained individuals that potentially can be accessed to voluntarily assist in interventions, first aid, or support line EMS in life threatening situations in the community. Training undertaken by health care professionals is ongoing, recurrent and offers a viable avenue to champion the concept of volunteer response within the community. The Resuscitation Council (UK) train large cohorts of health care professionals in life support courses, each having a different level of complexity. ILS and ALS are two such courses and considering the volume of health care professionals trained in both, it would be logical to hypothesise that survival to hospital discharge of OHCAs could improve due to an increase in trained citizens and early interventions. This does not seem to be the case, as evidenced in England; the national average of survival to hospital discharge is 7.9% from 2013 to 2018 despite having a yearly increase in the numbers of health care professionals trained in ILS and ALS [3, 13]. Creating options to mobilise these trained citizens is a potential next step and the technology available may offer means of improving access to these valuable resources.

Complex interventions such as novel crowdsourcing tools have been implemented over the last number of years across the globe to aid in improving access to volunteer first responders who can commence CPR and defibrillate before the arrival of EMS such as Pulse Point [14], GoodSAM [15] and SMS Lifesaver [16]. In the UK alone, GoodSAM have integrated their crowdsourcing technology with ambulance services in England, Wales and Northern Ireland allowing these services autonomy in alerting responders to OHCAs which is parallel to an EMS dispatch. These services are building capacity to improve the initial ‘call for help’ and time to commence CPR and defibrillation if indicated.

With the potential increased provision of skilled intervention in OHCAs comes the potential increased negative impacts on those responding and their wider family network. Recent years have seen a growing body of knowledge around the ‘work-family conflict crossover’ evidencing that the sometimes traumatic experiences of responders can have negative impacts within a responder’s family as a result of voluntary activities [17]. There is therefore a need to ensure both responders and their families are adequately supported.

## How to improve this state of affairs

There are just over 92,000 health care professionals trained in ILS or ALS and over 238,000 members of the public trained in CPR through Restart a Heart Day. This is not considering all other organisations and avenues for CPR and defibrillation training. Why then have survival rates not increased accordingly? Having the skills to deliver CPR and defibrillate are one key factor in improving survival. Getting to the scene of an OHCA to deliver those skills is another, and one which could potentially produce a huge impact on survival. New technologies have the potential to play a major role in linking these resources together and deliver them to the scene of an OHCA by ‘digitising the call for help’ [18].

Crowdsourcing technologies will likely have a key role to play in improving the ‘Chain of Survival’. By linking the local EMS with lay responders and health care professionals trained in CPR and defibrillation, a network of volunteer responders can be created to offer immediate and skilled intervention. The next step is to identify and implement appropriate methods for public engagement, involvement and eventual networking of resources with statutory bodies such as local EMS.

## Conclusions

There are large volumes of highly trained individuals across the UK - the ‘unknown network’ of responders who are not mobilised and who have not been engaged. Crowdsourcing has the potential to harness and optimise this talent. However, crowdsourcing volunteer responders is at an early stage and there is a need for robust research to determine: i) its association with patient outcomes; ii) its impact on those responding to OHCA; iii) whether it facilitates or impedes current services; and iv) whether it is a safe and cost effective way to involve citizens to intervene in the community during cardiac arrest or other medical emergencies. Addressing these issues will provide further insight into the role and effectiveness of new technologies and their potential impact on the wider community.

## Data Availability

Not applicable.
